# Enhanced stable production of ethylene in photosynthetic cyanobacterium *Synechococcus elongatus* PCC 7942

**DOI:** 10.1007/s11274-019-2652-7

**Published:** 2019-05-08

**Authors:** Veronica Carbonell, Eerika Vuorio, Eva-Mari Aro, Pauli Kallio

**Affiliations:** Molecular Plant Biology, Department of Biochemistry, University of Turku, 20014 Turun yliopisto, Finland

**Keywords:** Ethylene, Cyanobacteria, *Synechococcus elongatus* PCC 7942, Genetic stability, Photoautotrophic production, Biotechnological application

## Abstract

Ethylene is a volatile alkene which is used in large commercial scale as a precursor in plastic industry, and is currently derived from petroleum refinement. As an alternative production strategy, photoautotrophic cyanobacteria *Synechocystis* sp. PCC 6803 and *Synechococcus elongatus* PCC 7942 have been previously evaluated as potential biotechnological hosts for producing ethylene directly from CO_2_, by the over-expression of ethylene forming enzyme (efe) from *Pseudomonas syringae*. This work addresses various open questions related to the use of *Synechococcus* as the engineering target, and demonstrates long-term ethylene production at rates reaching 140 µL L^−1^ h^−1^ OD_750_^−1^ without loss of host vitality or capacity to produce ethylene. The results imply that the genetic instability observed earlier may be associated with the expression strategies, rather than efe over-expression, ethylene toxicity or the depletion of 2-oxoglutarate—derived cellular precursors in *Synechococcus*. In context with literature, this study underlines the critical differences in expression system design in the alternative hosts, and confirms *Synechococcus* as a suitable parallel host for further engineering.

**Electronic supplementary material:**

The online version of this article (10.1007/s11274-019-2652-7) contains supplementary material, which is available to authorized users.

## Introduction

Ethylene (C_2_H_4_) is a simple alkene which is widely used in chemical industry as a precursor for polymer synthesis and in food industry to induce fruit ripening. In addition, ethylene is a potential fuel with high energy density and other physicochemical properties suitable, for example, to combustion engines (Zulkarnain Abdul Latiff et al. 2008). The global ethylene demand is higher than 150 million tonnes per year (Petrochemical 2015) and it is primarily derived from non-renewable sources as a product in petroleum refining. The production of one ton of ethylene in the commonly used steam cracking process releases 1,5–3 tons of carbon dioxide in the atmosphere, which makes this one of the largest single CO_2_ emitting processes in chemical industry (Ungerer et al. 2012) and thus a significant global environmental burden.

In nature, ethylene has several distinct biological functions. In plants, it acts as a hormone associated with fruit ripening and abscission of leaves, and is produced from 1-aminocycloprone-1-carboxylate (ACC) by the enzyme ACC oxidase (Dong et al. 1992). Micro-organisms use ethylene, for example, in non-specific defence signalling (Gottwald et al. 2012) and as a mediator in virulence (Weingart et al. 2001), and it is produced via at least two different pathways: Ethylene biosynthesis may proceed through 2-keto-4-methylthiobutyric acid by the action of NADH:Fe(III)EDTA oxidoreductase as in *Cryptococcus albidus* (Fukuda et al. 1989; Ogawa et al. 1990)*,* or it can be generated from 2-oxoglutarate and l-arginine by ethylene forming enzyme (*efe*) as in various *Pseudomonas* species (Fukuda et al. 1986; Nagahama et al. 1991).

There is an increasing global need to develop and evaluate new solutions for the production of sustainable substitutes for petroleum-derived products such as ethylene. One of the potential biotechnological approaches is to use photosynthetic microbial cells, cyanobacteria, as engineered biological factories to produce different end-products of interest. This would allow the direct utilization of atmospheric CO_2_ and water as substrates for the biosynthesis of the target metabolites using sunlight as the sole source of energy, thus bypassing the use of biomass as starting material. In this respect, cyanobacteria have been extensively studied as engineering targets, and a range of molecular biology tools and production strategies have been developed and characterized [See reviews (Hagemann and Hess 2018; Sun et al. 2018)]. Besides ethylene, cyanobacteria have been engineered to produce various products, including alcohols, organic acids, and carbohydrates [see reviews (Oliver et al. 2016; Zhou et al. 2016)], but the overall efficiencies are still below the threshold required for any commercial applications, and require further systematic research.

Autotrophic production of ethylene has been studied primarily in two cyanobacterial strains S*ynechocystis* sp PCC 6803 (Guerrero et al. 2012; Ungerer et al. 2012; Eckert et al. 2014; Zhu et al. 2015; Lee et al. 2015; Xiong et al. 2015; Zavřel et al. 2016; Carbonell et al. 2016) and *Synechococcus elongatus* PCC 7942 (Fukuda et al. 1994; Sakai et al. 1997; Wang et al. 1999, 2000; Matsuoka et al. 2001; Takahama et al. 2003) (from here on referred to as *Synechocystis* and *Synechococcus*, respectively). The general strategy has been to over-express the heterologous ethylene forming enzyme from *Pseudomonas syringae* to convert endogenous metabolic precursors 2-oxoglutarate and l-arginine to ethylene, which then as a volatile gas spontaneously diffuses out from the cell and separates into the culture headspace.

In comparison to *Synechocystis*, only relatively low productivities have been achieved in stable *Synechococcus* strains (Supplementary Table S1), and the most efficient expression systems have been associated with instability and eventual loss of ethylene production in a few generations (Sakai et al. 1997; Takahama et al. 2003). The reported instability has been accompanied by apparent metabolic stress on the *Synechococcus* host, observed as decreased growth rates and chlorophyll breakdown resulting in a yellowish-green phenotype (Sakai et al. 1997; Takahama et al. 2003). At genetic level, the inactivation has been associated with insertion mutations taking place at specific repeated sequence elements (CGATG) which cause frameshifts in the *efe* gene (Takahama et al. 2003).

The aim of this study was to clarify different factors previously associated with the instability of the ethylene production systems in *Synechococcus*. Specifically, the intention was to (1) elucidate the role of the *efe* primary sequence in context with the chromosomal integration site, and (2) analyse possible stress effects caused by efe over-expression and ethylene levels, in order to obtain a more comprehensive view of the potential limiting factors in further developing *Synechococcus* as a platform for ethylene biosynthesis.

## Materials and methods

### Cell strains and default culture conditions

*Escherichia coli* strain DH5α was used for the molecular cloning steps and plasmid propagation. The cells were cultured in Luria–Bertani medium supplemented with 25 µg mL^−1^ of spectinomycin and 10 µg mL^−1^ of streptomycin (37 °C, 120 rpm shaking). *Synechococcus elongatus* PCC 7942 was used as the efe over-expression host for ethylene production. The cells were cultivated in BG11 liquid medium (20 mM TES-KOH, pH 8) supplemented with 25 µg mL^−1^ of spectinomycin, 10 µg mL^−1^ of streptomycin and 1 mM IPTG (Geerts et al. 1995; Berla et al. 2013) when appropriate. The cultures were carried out in 100 mL volume (250 mL Erlenmeyer flasks) under continuous light (50 µmol photons m^−2^ s^−1^) at 30 °C in 1% CO_2_ in an orbital shaker (120 rpm) in a Sanyo Chamber (SanyoElectric Co. Ltd).

### Assembly of the efe over-expression constructs and transformation into *Synechococcus*

**Fig. 1:**
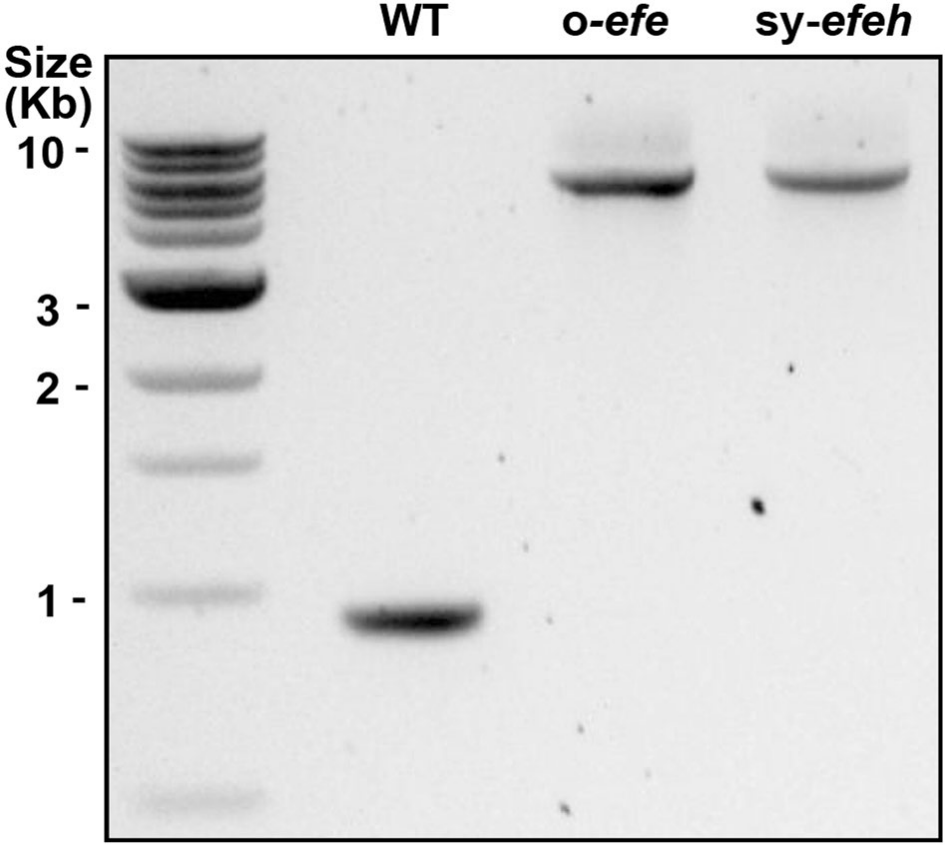
Colony PCR (1% agarose gel) confirming chromosomal integration and segregation of the *efe* expression cassettes at the *NSI*-locus of *Synechococcus elongatus* PCC 7942. Wild type control (WT; expected fragment size = 906 bp), *Synechococcus* carrying *efe* from *P. Syringae* (o-*efe*; expected fragment size = 5446 bp), and *Synechococcus* carrying sequence optimized *efe* (sy-*efeh*; expected fragment size = 5446 bp). The *NSI*-specific primers used for the colony PCR are listed in Table 2

The commercial plasmid pUC19 (Yanisch-Perron et al. 1985) was used as the backbone to assemble a chromosomal integration vector for the introduction of *efe* in *Synechococcus* (Table 1). A 1094 bp fragment allowing targeted homologous recombination into the host genome at the *NSI* site (GenBank: U30252.3) (Mackey et al. 2007) was PCR amplified (Table 2, primers 1 and 2) and subcloned (AatII-BamHI) into pUC19 to create p19_7942_NSI (Table 1). Subsequently, two *efe* gene variants, o-*efe* (GenBank: D13182.1) and sy-*efeh* (GenBank: KX184731)*,* were PCR amplified (Table 2, primers 3 and 4) from pDF-trc-o-EFE (Carbonell et al. 2016) and pDF-trc-EFEh (Guerrero et al. 2012), respectively, to create fragments carrying a spectinomycin/streptomycin resistance cassette (Sp^r^/Str^r^), *lac* repressor (*LacI*^*q*^), *trc* promoter (P_*trc*_) and the two *rrnB* terminators. The fragments were inserted into p19_7942_NSI (Eagl) creating the final integration constructs pChr_7942_NSI_o-*efe*_Sp and pChr_7942_NSI_sy-efeh_Sp (Table 1), which were then confirmed by sequencing (Table 2, primers 5–8). The plasmids were transformed into *Synechococcus* via natural transformation and the resulting recombinant strains were called *Synechococcus*:o-*efe* and *Synechococcus*:sy-*efeh* respectively. Full segregation of the mutants at the *NSI* locus was verified by PCR (Fig. 1).

**Table 1:** Plasmids used in the study

Plasmids	Description	References
pUC19	Ap^r^, ori (ColE1)	(Yanisch-Perron et al. 1985)
p19_7942_NSI	pUC19 derivative containing 1,1 kb fragment of *NSI* from *Synechococcus*	This study
pDF-trc-EFEh	sy-*efeh* gene, *rrnB* terminator regions and Sp^r^/Str^r^ cassette for insertion into p19_7942_*NSI*	(Guerrero et al. 2012)
pDF-trc-o-EFE	o-*efe* gene, *rrnB* terminator regions and Sp/Str cassette for insertion into p19_7942_*NSI*	(Carbonell et al. 2016)
pChr_7942_NSI_o-efe_Sp	Derivative p19_7942_*NSI* containing o-*efe* gene, rrnB terminator regions and Sp^r^/Str^r^ cassette flanked by *NSI*	This study
pChr_7942_NSI_sy-efeh_Sp	Derivative p19_7942_*NSI* containing sy-*efeh* gene, *rrnB* terminator regions and Sp^r^/Str^r^ cassette flanked by *NSI*	This study

**Table 2:** PCR Primers (5′→3′ direction) used in this study

ID	Primers	Sequence and description
1	fw_SEA0027	attagacgtcTAGTCGCCGCAGTAGTGATG Cloning *NSI* from *Synechococcus* genome (AatII overhang)
2	rv_SEA0027	aatggatccACCCGGTAGGGATTTCG Cloning *NSI* from *Synechococcus* genome (BamHI overhang)
3	fw_pDF_s_iq_e_t2	CTGGCTTTGCTTCCAGATGT Cloning of pDF cassette containing Sp^r^/Str^r^, *LacI*^*q*^, P_*trc*_, *efe* variants and two *rrnB* terminators
4	rv_pDF_s_iq_e_t2_EagI	taaacggccgCTTTCAGCTAGCGTACCA Cloning of pDF cassette containing Sp^r^/Str^r^, *LacI*^*q*^, P_*trc*_, *efe* variants and two *rrnB* terminators (Eagl overhang)
5	fw_seq_NSI_7942	TAGTCGCCGCAGTAGTGATG Sequencing and colony PCR
6	rv_seq_NSI_7942	CTCCAGCAAGCTAGCGATTT Sequencing and colony PCR
7	75_pUC_Rev	GCTCACTCATTAGGCACCCCAGG Sequencing and colony PCR
8	rv_seq_NSI_ins	AGGGCCGTGATCTTGTCAT Sequencing and colony PCR

Complementary regions are shown in capital letters and restriction sites are underlined

### Quantitative analysis of ethylene production

Ethylene production of the strains was monitored in a step-wise experiment of four repeated consecutive 100 mL cultivation batches, with four biological replicates each. Every cultivation batch was started at OD_750_ ~ 0.1 and grown until the optical density reached ~ 1 (after about 4 days), followed by analysis of ethylene production and inoculation of the successive batch. Ethylene quantitation was carried out by transferring 1 mL of the cell cultures supplemented with 1 mM IPTG into 10 mL sealed vials, followed by 2–8 h incubation under specified conditions (50 µmol photons m^−2^ s^−1^, 30 °C, 120 rpm), and analysis of the headspace gas phase (25–250 µL samples) by GC–MS as described earlier (Guerrero et al. 2012; Carbonell et al. 2016). The integrated MS peak areas were used to calculate the relative ethylene production as µL of ethylene per litre of culture per hour (µL L^−1^ h^−1^), and normalized to cell density to allow comparison. The statistical significance of the observed differences was evaluated based on the calculated average values, using pairwise *t*-test analysis at significance level *p* ≤ 0.05.

### Evaluation of expression system stability in long-term cultivations

Long-term stability of the *Synechococcus* ethylene production strains was evaluated in a 16-week step-wise batch cultivation trial, carried out in 40 mL culture volume in 100 mL Erlenmeyer flasks in four parallel replicates. The cultures were diluted in fresh BG11 (containing antibiotics) to OD_750_ 0.05 at 1 week intervals, and analysed for ethylene productivity after 7 days incubation.

### Effect of supplemented ethylene on cell growth

The effect of high concentrations of ethylene on WT *Synechococcus* was evaluated by supplying 99% (v/v) gaseous ethylene (AGA, Espoo, Finland) into 20 mL cell cultures containing 50 mM bicarbonate in gas-tight serum bottles (160 mL). Ethylene was supplied directly into the culture media by bubbling for 2 min (≈ 0.5 bars) with an injection needle through the butyl rubber cap of the sealed bottles (equipped with an additional outlet needle to prevent the build-up of overpressure). Air and nitrogen gas were used as parallel controls. Growth of the cells was followed by monitoring culture OD_750_ for the subsequent 7 days.

### Substrate supplementation

The quantitative effect of substrate supplementation on ethylene production was evaluated by the addition of 5 mM l-arginine (Sigma-Aldrich) or/and 1 mM 2-oxoglutarate (Sigma-Aldrich) into the sealed 10 ml reaction vials at the same time with IPTG. The analysis was carried out in four biological replicates, and the ethylene productivity was compared against corresponding reactions without supplemented substrates.

### Spectral analysis of the cultures

Absorption spectra of the *Synechococcus* cultures (400–750 nm) were recorded with Infinite 200 Pro plate reader (Tecan Ltd, Switzerland). The spectra were obtained from 150 µL of cell cultures normalized to the same OD_750_ on 96 well plates (Greiner 96 Flat Bottom Transparent Polystyrol).

## Results

### Construction of *Synechococcus* strains for ethylene production

In order to evaluate the production of ethylene in *Synechococcus*, two chromosomal integration constructs were assembled for the over-expression of the heterologous ethylene forming enzyme under the control of a constitutive promoter *P*_*trc*_ (Huang et al. 2010; Guerrero et al. 2012). Two alternative forms of the *efe* gene used in the constructs were (i) the native *efe* from *P. Syringae* (o-*efe*) used in several earlier studies in *Synechococcus* (Fukuda et al. 1994; Sakai et al. 1997; Takahama et al. 2003) and (ii) a sequence-optimized form (sy-*efeh*) previously expressed in *Synechocystis*, from which the repetitive sequences CGATG associated with insertion mutations had been removed (Ungerer et al. 2012; Guerrero et al. 2012; Carbonell et al. 2016). The chromosomal locus *NSI* was selected as the target site for homologous recombination, as it had previously successfully used for protein over-expression in *Synechococcus* (Ditty et al. 2005; Mackey et al. 2007). The sequenced constructs were transformed into *Synechococcus*, followed by selection based on acquired antibiotic resistance. Colony PCR was used to confirm integration at the *NSI* site and full segregation of the generated mutants (Fig. 1), and in each case, only fragments corresponding to the expected size (5446 bp) were observed while the WT fragment was not detected (906 bp).

### The two generated *Synechococcus* strains produce ethylene at similar levels

**Fig. 2:**
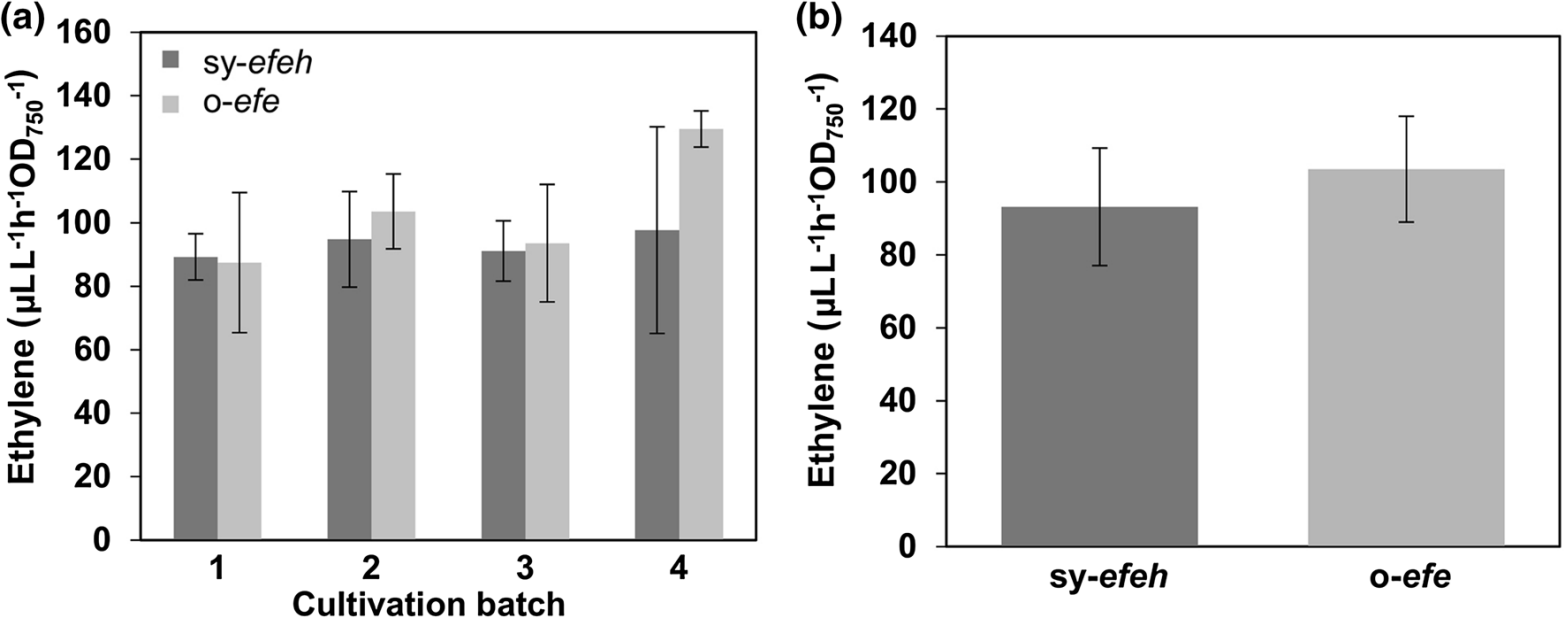
Ethylene production by *Synechococcus* *elongatus* PCC 7942 over-expressing sy-*efeh* and o-*efe* in four successive 4-day batch cultures. **a** Production rate averages of the four batches normalized to OD_750_ (n = 4; error bars represent SD). **b** Average ethylene production throughout the entire experiment (n = 16; error bars represent SD). Statistically significant differences were not observed between the cultivation batches or the alternative strains (*t* test)

Cultivation of both of the generated *Synechococcus* efe over-expression strains resulted in the accumulation of ethylene in the headspace of the closed culture vials as detected by GC–MS. The production efficiency of the two strains was very similar, and no obvious difference could be observed between the function of the o-*efe* or the *sy-efeh* under the conditions tested (Fig. 2a). In both cases the ethylene productivity remained stable throughout the four consecutive 4-day batch cultures, with highest recorded yields of around 140 µL L^−1^ h^−1^ OD_750_^−1^, averaging to about 100 µL L^−1^ h^−1^ OD_750_^−1^ (Fig. 2b).

### Expression of efe or direct ethylene supplementation do not induce adverse effects to the cells

**Fig. 3:**
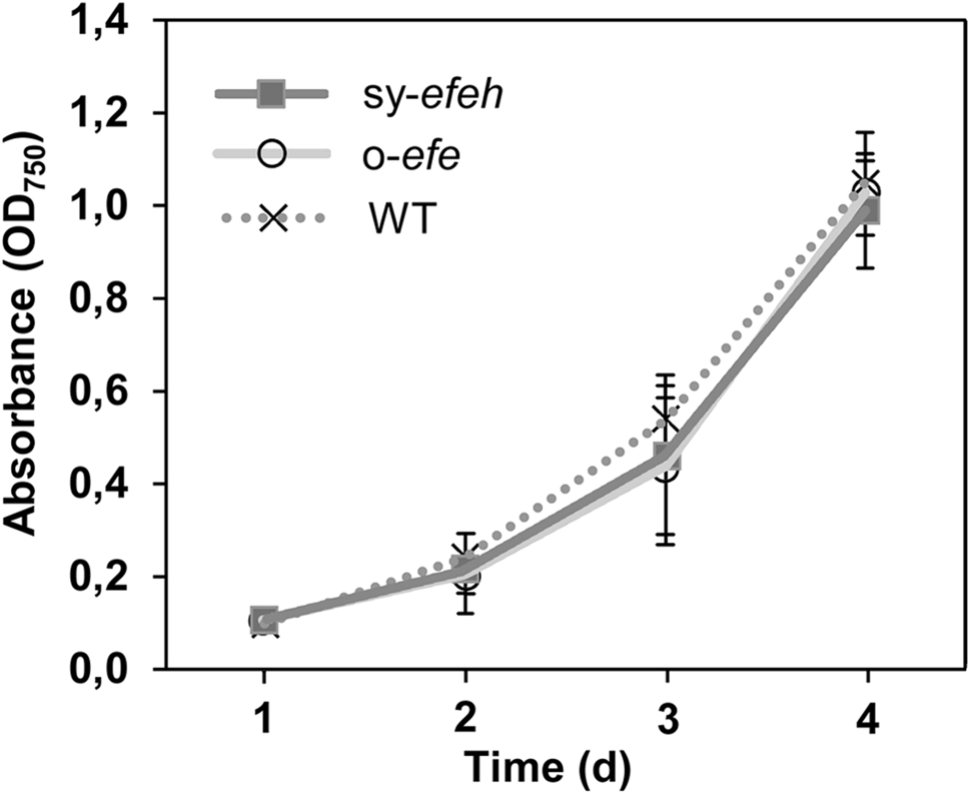
Growth (OD_750_) of *Synechococcus elongatus* PCC 7942 wild type (dotted line), *Synechococcus*:o-*efe* (solid light grey line) and *Synechococcus*:sy-*efeh* (solid dark grey line) in the successive 4-day batch cultures (n = 3, error bars represent SD). Statistically significant differences were not observed between the growth of the alternative strains (*t* test)

**Fig. 4:**
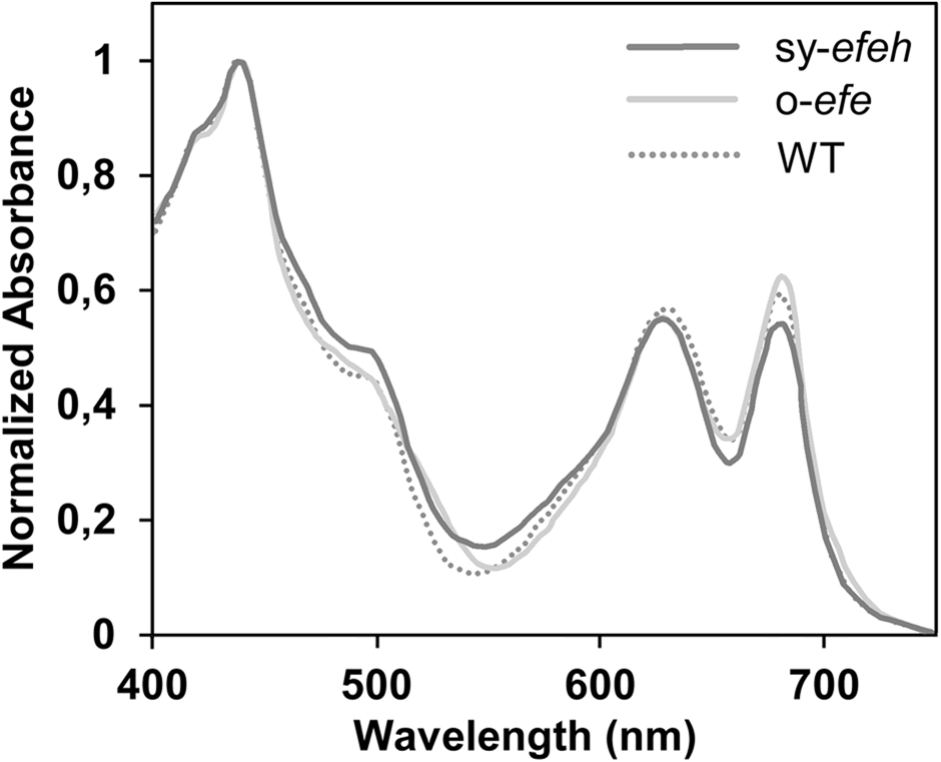
Absorption spectra of *Synechococcus elongatus* PCC 7942 wild type (dotted line), *Synechococcus*:o-*efe* (solid light grey line) and *Synechococcus*:sy-*efeh* (solid dark grey  line). Absorption curves were obtained from three replicates of each strain and normalized to OD_750_

**Fig. 5:**
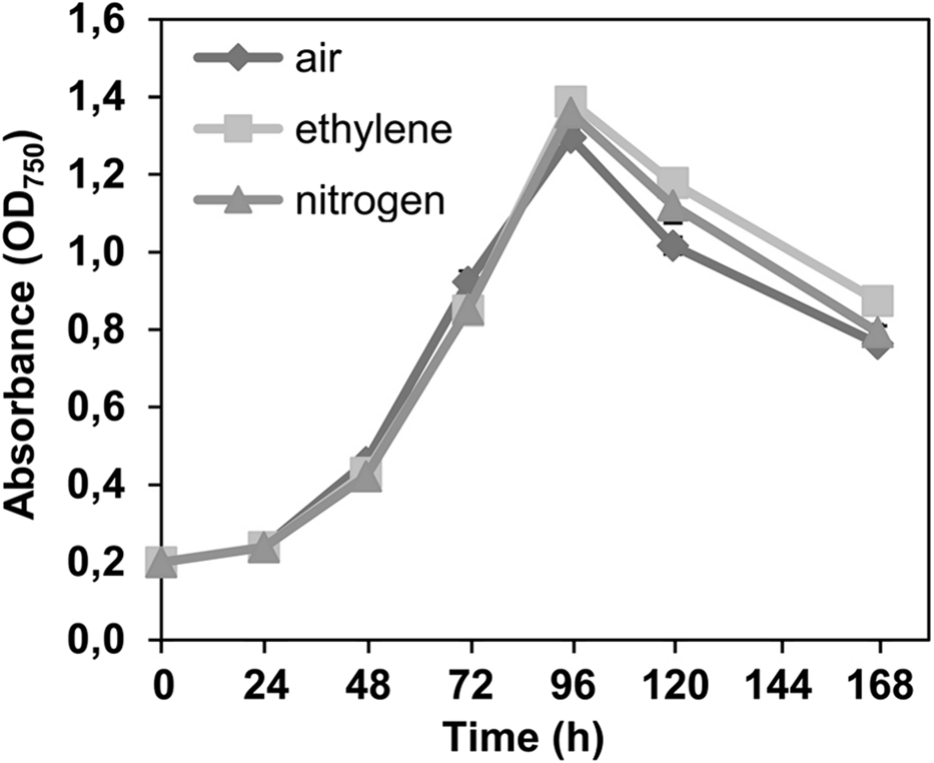
Evaluation of the tolerance of *Synechococcus elongatus* PCC 7942 wild type towards supplemented ethylene. The cell cultures (20 mL) with 50 mM of bicarbonate were incubated for 7 days in sealed 160 mL serum bottles under ethylene atmosphere (squares). As controls, parallel cultures were incubated with air (diamonds) and with pure nitrogen (triangles). (n = 3; error bars represent SD). The decline in growth after 96 h is primarily due to the depletion of bicarbonate from the air-tight cultivation flasks. Statistically significant differences were not observed in cell growth between the alternative culture conditions (*t* test, *p* ≤ 0.05)

With the objective to evaluate possible adverse effects caused by ethylene production to the host, the two efe-expressing strains were compared against the wild type *Synechococcus* strain. The results showed that constitutive ethylene production at the recorded levels did not have any clear negative impact on the growth of the host; The growth rates of all the strains were comparable (Fig. 3), and the phenotype based on culture colour and absorption properties measured spectrophotometrically (Fig. 4) were similar. Furthermore, the presence of high concentrations of supplied ethylene (up to > 99% of the headspace gas) in sealed 7-day batch cultures did not affect the viability of the cells, indicating that ethylene itself does not inflict any acute toxicity effects on *Synechococcus* even at saturating concentrations (Fig. 5).

### The engineered strains maintain the capacity to produce ethylene in long-term cultivation

**Fig. 6:**
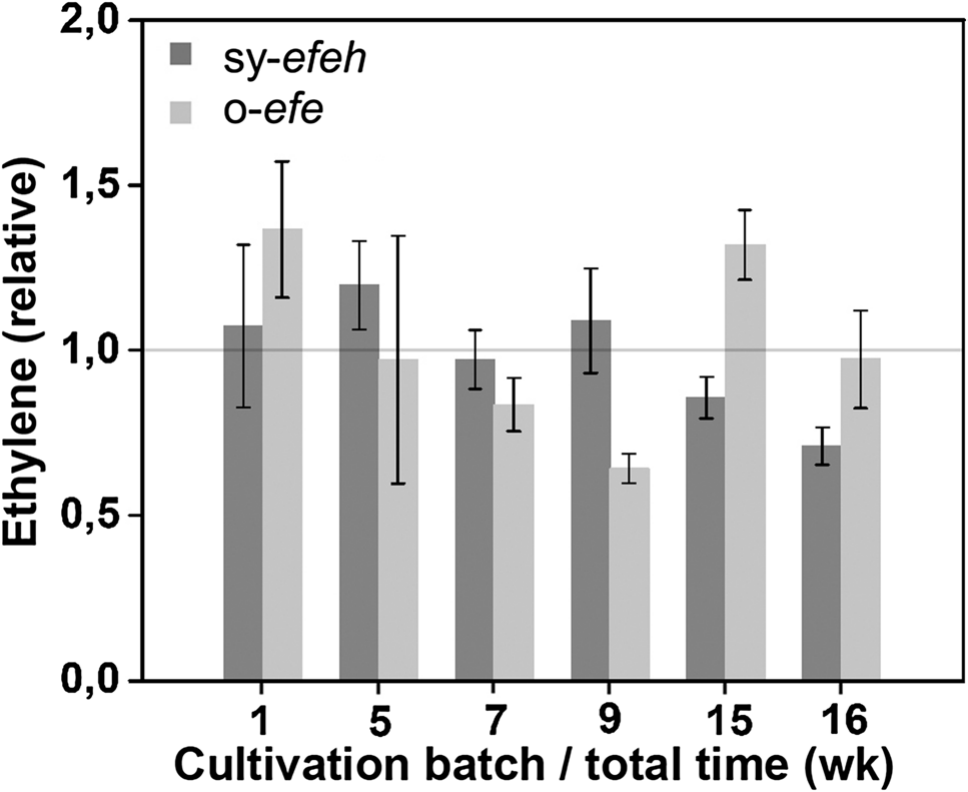
Relative long-term ethylene productivity recorded for *Synechococcus elongatus* PCC 7942 sy-*efeh* and o-*efe* over-expression strains in successive batch cultures over a period of 16 weeks. The mean and standard deviations represent four biological replicates, and the data is normalized to the total calculated average (grey horizontal line). The relatively large fluctuation between the successive measurement time points (*cf.* Data in Figs. 2, 7) is due to a different experimental set-up with smaller cultures and extended incubation time between dilution and the measurement (7 days), in which minor variations in cultivation conditions have a proportionally significant effect on the recorded numerical values

In order to further evaluate the stability of the *Synechococcus* ethylene-producing strains in long-term incubation, the cultures were subjected to a 16-week step-wise batch cultivation, in which the cultures were diluted once a week in fresh BG11 medium and analysed for productivity at given time-points (Fig. 6). The results clearly demonstrated that, despite some batch-specific fluctuation, the overall capacity to produce ethylene was not lost nor significantly reduced in over 3-month expression trials.

### Supplementation of efe substrates does not significantly improve productivity

**Fig. 7:**
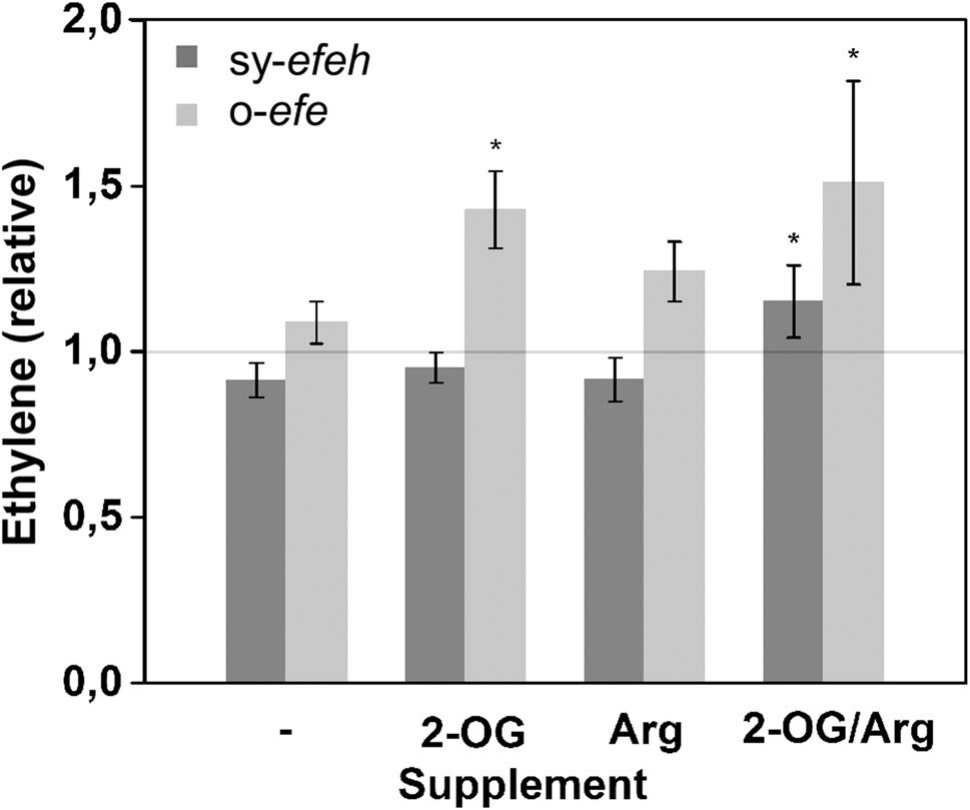
Evaluation of the effect of supplemented substrates, 2-oxoglutarate (1 mM) or/and l-arginine (5 mM) on relative ethylene productivity in *Synechococcus elongatus* PCC 7942 sy-*efeh* and o-*efe* over-expression strains. The data is normalized to the calculated average of the non-supplemented strains (grey horizontal line). The mean and standard deviations represent four biological replicates. The asterisk indicates statistically significant change (*t* test, *p* ≤ 0.05) in reference to the corresponding control without supplemented substrates

To obtain more information on the key factors limiting ethylene productivity of the engineered *Synechococcus* strains, the production cultures were incubated in the presence of the two primary substrates of efe, 2-oxoglutarate and l-arginine. Although the uptake of the compounds from the medium was expected to be at least partially limited for *Synechococcus* (Vázquez-Bermúdez et al. 2000; Montesinos et al. 1997) a clear increase in ethylene formation was observed for both strains supplemented with the two substrates (Fig. 7). However, this improvement was rather subtle, with average values remaining under 40% at best, and apparently susceptible to relatively minor alterations, as observed in the different response of the strains towards 2-oxoglutarate and l-Arginine when supplied one at a time.

## Discussion

Ethylene is a prominent target for the development of novel biotechnological applications due to the large global market, and need for sustainable alternatives for the current petroleum-based technologies. This study focused on engineered photoautotrophic cyanobacterial systems that allow the production of ethylene directly from CO_2_, with a specific focus of critical strain-specific constraints associated with using *Synechococcus* as the host. Majority of earlier studies have focused on another cyanobacterial species *Synechocystis*, which has generally exhibited higher ethylene production levels and stability (Supplementary Table S1). In comparison, apart from systems with very low productivities that have been apparently stable, *efe* over-expression in *Synechococcus* has repeatedly been associated with metabolic stress and compromised expression system stability observed as decreased efficiency and eventual loss of ethylene production (Sakai et al. 1997; Takahama et al. 2003) (Supplementary Table S1). With the objective of evaluating the potential for further development, this study compared the activity of two alternative forms of the *efe* gene in *Synechococcu*s, and addressed (i) the effect of the site of chromosomal integration and (ii) the impact of ethylene levels on the stability and performance of the production system.

Two different variants of *efe* (native gene from *P. Syringae* and a codon optimized sequence) were inserted at the *NSI* locus (Ditty et al. 2005) in *Synechococcus* chromosome under the regulation of *trc* promoter (Huang et al. 2010; Guerrero et al. 2012). Both expression systems were functional, producing in average around 100µL L^−1^ h^−1^ OD_750_^−1^ ethylene (Fig. 2), without apparent loss of the capacity in extended cultivations (Fig. 6). Notably, the maximum production levels (approaching 140 µL L^−1^ h^−1^ OD_750_^−1^) were almost three-fold higher than earlier reported for any stable ethylene production system in *Synechococcus* (Sakai et al. 1997) (Supplementary Table S1), providing a starting point for comparing and evaluating the possible factors behind the problems encountered earlier. It must be emphasized that while the numbers are still somewhat lower than recorded for corresponding *Synechocystis* strains, and significantly below the highest reported values in the most extensively engineered systems (Mo et al. 2017), the baseline for ethylene production appears to be rather similar for the two strains.

The only form of *efe* that has previously been expressed in *Synechococcus* is the native gene form *P. Syringae*, which has been reported to accumulate insertion mutations resulting in *efe* inactivation and loss productivity in several generations (Takahama et al. 2003). Our observation that both expressed forms of *efe* remained equally functional in *Synechococcus* (Figs. 2, 3, 4) confirmed the expectation that the nucleotide sequence *per se* does not play any critical role in determining system integrity. Comparison of the different expression strategies used in *Synechococcus* (Supplementary Table S1), however, reveals that the unstable systems specifically apply *psbAI* locus for expression cassette integration (Takahama et al. 2003), or contain sequence elements (promoter and terminator sequences) which may promote homologous recombination at this site (Sakai et al. 1997). It is conceivable that these approaches render the endogenous *Synechococcus* *psbAI* gene inactive and defective in the production of the specific photosystem II reaction center protein D1, which is essential under normal growth conditions (Golden et al. 1986; Aro et al. 1993). It is important to note here that the expression of alternative *psbA* genes in response to environmental cues, as well as the function of the corresponding D1 proteins, are critically different in *Synechococcus* and *Synechocystis* (Mulo et al. 2009). This explains why *psbAI* in *Synechocystis*, unlike in *Synechococcus*, can be disrupted without compromising viability (Varman et al. 2013; Yu et al. 2013), and emphasizes the crucial importance of taking host-specific features into account when selecting the expression strategy for a particular organism.

In regards to acute toxicity, the study confirms that ethylene in itself does not induce any detectable adverse effects on the growth or viability of *Synechococcus* even when supplied at saturating concentrations in prolonged incubation (Fig. 5). Thus, interference of ethylene in native metabolic functions would not pose limitations for using *Synechococcus* as a host for biotechnological applications. One of the bottlenecks which have been proposed for ethylene biosynthesis, however, is the depletion of 2-oxoglutarate—derived building blocks in the cell (Takahama et al. 2003) that would compromise a wide range of native metabolic activities including biosynthesis of proteins and nucleic acids. Despite production levels which were comparable with earlier reports (Sakai et al. 1997), such adverse effects were not observed in the current study, and the host cells appeared not to suffer from biosynthetic burden resulting from the shortage of 2-oxoglutarate. In addition, even though the uptake of extracellular substrates is likely to be at least partly restricted by the diffusion through the membrane, the relatively moderate effect observed for 2-oxoglutarate and l-arginine supplementation on ethylene production (Fig. 7) reinforces the view that also other factors besides substrate limitation currently constraint the system.

Our conclusion is that the key limiting factors in using *Synechococcus* as a host for photoautotrophic production of ethylene are not related to adverse effects caused efe over-expression or the presence of ethylene. Instead, the biosynthetic bottlenecks may be similar to those identified for *Synechocystis*: (i) Restricted overall flux towards the TCA cycle, and (ii) strictly regulated feedback inhibition of the three first catalytic steps of the cycle including conversion of isocitrate to 2-oxoglutarate, which could be reinforced by (iii) enhancing the expression of efe to increase the efficiency of ethylene formation, thus increasing the biosynthetic pull throughout the pathway.

## Electronic supplementary material

Below is the link to the electronic supplementary material.

Supplementary file1 (PDF 104 kb)

## References

[CR1] Aro EM, Virgin I, Andersson B (1993). Photoinhibition of photosystem. II. Inactivation, protein damage and turnover. Biochim Biophys Acta.

[CR2] Berla BM, Saha R, Immethun CM, Maranas CD, Moon TS, Pakrasi HB (2013). Synthetic biology of cyanobacteria: unique challenges and opportunities. Front Microbiol.

[CR3] Carbonell V, Vuorio E, Aro E-M, Kallio P (2016). Sequence optimization of efe gene from *P. syringae* is not required for stable ethylene production in recombinant *Synechocystis* sp. PCC 6803. IJIRTS.

[CR4] Ditty JL, Canales SR, Anderson BE, Williams SB, Golden SS (2005). Stability of the *Synechococcus elongatus* PCC 7942 circadian clock under directed anti-phase expression of the kai genes. Microbiology.

[CR5] Dong JG, Fernández-Maculet JC, Yang SF (1992). Purification and characterization of 1-aminocyclopropane-1-carboxylate oxidase from apple fruit. PNAS.

[CR6] Eckert C, Xu W, Xiong W, Lynch S, Ungerer J, Tao L, Gill R, Maness PC, Yu J (2014). Ethylene-forming enzyme and bioethylene production. Biotechnol Biofuels.

[CR7] Fukuda H, Fujii T, Ogawa T (1986). Preparation of a cell-free ethylene-forming system from *Penicillium digitatum*. Agric Biol Chem.

[CR8] Fukuda H, Takahashi M, Fujii T, Tazaki M, Ogawa T (1989). An NADH:Fe(III)EDTA oxidoreductase from *Cryptococcus albidus*: an enzyme involved in ethylene production in vivo?. FEMS Microbiol Lett.

[CR9] Fukuda H, Sakai M, Nagahama K, Fujii T, Matsuoka M, Inoue Y, Ogawa T (1994). Heterologous expression of the gene for the ethylene-forming enzyme from *Pseudomonas Syringae* in the cyanobacterium *Synechococcus*. Biotechnol Lett.

[CR10] Geerts D, Bovy A, de Vrieze G, Borrias M, Weisbeek P (1995). Inducible expression of heterologous genes targeted to a chromosomal platform in the cyanobacterium *Synechococcus* sp. PCC 7942. Microbiology.

[CR11] Golden SS, Brusslan J, Haselkorn R (1986). Expression of a family of psbA genes encoding a photosystem II polypeptide in the cyanobacterium *Anacystis nidulans* R2. EMBO J.

[CR12] Gottwald S, Samans B, Lück S, Friedt W (2012). Jasmonate and ethylene dependent defence gene expression and suppression of fungal virulence factors: two essential mechanisms of *Fusarium* head blight resistance in wheat?. BMC Genomics.

[CR13] Guerrero F, Carbonell V, Cossu M, Correddu D, Jones PR (2012). Ethylene synthesis and regulated expression of recombinant protein in *Synechocystis* sp. PCC 6803. PLoS ONE.

[CR14] Hagemann M, Hess WR (2018). Systems and synthetic biology for the biotechnological application of cyanobacteria. Curr Opin Biotechnol.

[CR15] Huang H-H, Camsund D, Lindblad P, Heidorn T (2010). Design and characterization of molecular tools for a synthetic biology approach towards developing cyanobacterial biotechnology. Nucleic Acids Res.

[CR16] Kuchmina E, Klähn S, Jakob A, Bigott W, Enke H, Dühring U, Wilde A (2017). Ethylene production in *Synechocystis* sp. PCC 6803 promotes phototactic movement. Microbiology.

[CR17] Latiff ZA, Aziz AA, Supriyo B, Said M (2008). Viability study of ethylene (C_2_H_4_) as an alternative fuel for gasoline engine. Jurnal Mekanikal.

[CR18] Lee TC, Xiong W, Paddock T, Carrieri D, Chang IF, Chiu HF, Ungerer J, Hank Juo SH, Maness PC, Yu J (2015). Engineered xylose utilization enhances bio-products productivity in the cyanobacterium *Synechocystis* sp. PCC 6803. Metab Eng.

[CR19] Mackey SR, Ditty JL, Clerico EM, Golden SS (2007). Detection of rhythmic bioluminescence from luciferase reporters in cyanobacteria. Methods Mol Biol.

[CR20] Matsuoka M, Kazutaka T, Takahira O (2001). Gene replacement in cyanobacteria mediated by a dominant streptomycin-sensitive rps12 gene that allows selection of mutants free from drug resistance markers. Microbiology.

[CR21] Mo H, Xie X, Zhu T, Lu X (2017). Effects of global transcription factor NtcA on photosynthetic production of ethylene in recombinant *Synechocystis* sp. PCC 6803. Biotechnol Biofuels.

[CR22] Montesinos ML, Herrero A, Flores E (1997). Amino acid transport in taxonomically diverse cyanobacteria and identification of two genes encoding elements of a neutral amino acid permease putatively involved in recapture of leaked hydrophobic amino acids. J Bacteriol.

[CR23] Mulo P, Sicora C, Aro EM (2009). Cyanobacterial psbA gene family: optimization of oxygenic photosynthesis. Cell Mol Life Sci.

[CR24] Nagahama K, Ogawa T, Fujii T, Tazaki M, Tanase S, Morino Y, Fukuda H (1991). Purification and properties of an ethylene-forming enzyme from *Pseudomonas syringae* pv. *phaseolicola* PK2. Microbiology.

[CR25] Ogawa T, Takahashi M, Fujii T, Tazaki M, Fukuda H (1990). The role of NADH:Fe(III)EDTA oxidoreductase in ethylene formation from 2-keto-4-methylthiobutyrate. J Ferment Bioeng.

[CR26] Oliver NJ, Rabinovitch-Deere CA, Carroll AL, Nozzi NE, Case AE, Atsumi S (2016). Cyanobacterial metabolic engineering for biofuel and chemical production. Curr Opin Chem Biol.

[CR27] Petrochemical (2015) The ethylene technology report. In: Research and markets, p 200

[CR28] Sakai M, Takahira O, Masayoshi M, Hideo F (1997). Photosynthetic conversion of carbon dioxide to ethylene by the recombinant cyanobacterium, *Synechococcus* sp. PCC 7942, which harbors a gene for the ethylene-forming enzyme of *Pseudomonas syringae*. J Ferment Bioeng.

[CR29] Sun T, Li S, Song X, Diao J, Chen L, Zhang W (2018). Toolboxes for cyanobacteria: recent advances and future direction. Biotechnol Adv.

[CR30] Takahama K, Masayoshi M, Kazuhiro N, Takahira O (2003). Construction and analysis of a recombinant cyanobacterium expressing a chromosomally inserted gene for an ethylene-forming enzyme at the psbAI locus. J Biosci Bioeng.

[CR31] Ungerer J, Ling T, Mark D, Maria G, Pin-Ching M, Jianping Y (2012). Sustained photosynthetic conversion of CO_2_ to ethylene in recombinant cyanobacterium *Synechocystis* 6803. Energy Environ Sci.

[CR32] Varman AM, Xiao Y, Pakrasi HB, Tang YJ (2013). Metabolic engineering of *Synechocystis* sp. strain PCC 6803 for isobutanol production. Appl Environ Microbiol.

[CR33] Vázquez-Bermúdez MF, Herrero A, Flores E (2000). Uptake of 2-oxoglutarate in *Synechococcus* strains transformed with the *Escherichia coli kgtP* gene. J Bacteriol.

[CR34] Veetil VP, Angermayr SA, Hellingwerf KJ (2017). Ethylene production with engineered *Synechocystis* sp PCC 6803 strains. Microb Cell Fact.

[CR35] Wang J, Araki T, Ogawa T, Matsuoka M, Fukuda H (1999). A method of graphically analyzing substrate-inhibition kinetics. Biotechnol Bioeng.

[CR36] Wang J-S, Araki T, Matsuoka M, Ogawa T (2000). A Model of photoinhibition related to mRNA instability in ethylene production by a recombinant cyanobacterium. J Theor Biol.

[CR37] Weingart H, Ullrich H, Geider K, Völksch B (2001). The role of ethylene production in virulence of *Pseudomonas syringae* pvs. *glycinea* and *phaseolicola*. Phytopathology.

[CR38] Xiong W, Morgan JA, Ungerer J, Wang B, Maness P-C, Yu J (2015). The plasticity of cyanobacterial metabolism supports direct CO_2_ conversion to ethylene. Nat Plants.

[CR39] Yanisch-Perron C, Vieira J, Messing J (1985). Improved Ml3 phage cloning vectors and host strains: nucleotide sequences of the M13mp18 and pUC19 vectors. Gene.

[CR40] Yu Y, You L, Liu D, Hollinshead W, Tang YJ, Zhang F (2013). Development of *Synechocystis* sp. PCC 6803 as a phototrophic cell factory. Mar Drugs.

[CR41] Zavřel T, Knoop H, Steuer R, Jones PR, Červený J, Trtílek M (2016). A quantitative evaluation of ethylene production in the recombinant cyanobacterium *Synechocystis* sp. PCC 6803 harboring the ethylene-forming enzyme by membrane inlet mass spectrometry. Bioresour Technol.

[CR42] Zhou J, Zhu T, Cai Z, Li Y (2016). From cyanochemicals to cyanofactories: a review and perspective. Microb Cell Fact.

[CR43] Zhu T, Xiaoman X, Zhimin L, Xiaoming T, Xuefeng L (2015). Enhancing photosynthetic production of ethylene in genetically engineered *Synechocystis* sp. PCC 6803. Green Chem.

