# Supplementary material for: Enhanced stable production of ethylene in photosynthetic cyanobacterium Synechococcus elongatus PCC 7942
Source: World J Microbiol Biotechnol. 2019 May 8;35(5):77. doi: 10.1007/s11274-019-2652-7 (PMC6536634; doi:10.1007/s11274-019-2652-7)
Supplement: Supplementary file 1 — Supplementary file1 (PDF 104 kb) [file 11274_2019_2652_MOESM1_ESM.pdf]

**Supplementary Table S1:** Comparison of different selected *efe* ethylene production systems constructed in *Synechococcus elongatus* PCC 7942 and *Synechocystis* sp. PCC 6803 in regards to reported efficiency and stability. The listed productivities are the highest reported values under the different cultivation conditions tested.

| ID | Host and other genetic modifications | *efe* gene and modifications | Expression plasmid/ integration site (number of *efe* genes) | Promoter | Max. reported productivity ( $\mu L*L^{-1}*h^{-1}*OD^{-1}$ ) | Stability | Phenotype | Reference |
|---|---|---|---|---|---|---|---|---|
| 1 | *Synechococcus* R2-SPc ΔpUH25 | original | pUC303-EFE03 | $P_{efe}$ | 25 | N.R. | N.R. | (Fukuda 1994) |
| 2 | *Synechococcus* R2-SPc ΔpUH24 | original | pUC303-EFE03 | $P_{efe}$ | 52,9 | + | blue-green | (Sakai et al. 1997) |
| 3 | *Synechococcus* R2-SPc ΔpUH24 | original | pUC303-EFE10 | $P_{lacZ}$ | 17,6 | + | blue-green | (Sakai et al. 1997) |
| 4 | *Synechococcus* R2-SPc ΔpUH24 | original | pUC303-EFE30 | $P_{lacZ}$ | 11,1 | + | blue-green | (Sakai et al. 1997) |
| 5 | *Synechococcus* R2-SPc ΔpUH24 | original | pUC303-pEXE3-I | $P_{psbA1}$ | 15,9 | - | green | (Sakai et al. 1997) |
| 6 | *Synechococcus* R2-SPc ΔpUH24 | original | pUC303-pEXE3 Δ1-Δ8 [$P_{psbA1}$ variations] | $P_{psbA1}$ | 130-323 | - | yellow-green | (Sakai et al. 1997) |
| 7 | *Synechococcus* R2-SPc ΔpUH24 | original | pUC303-pEXEΔ9 | $P_{psbA1}$ shrunken | 15 | + | blue-green | (Sakai et al. 1997) |
| 9 | *Synechococcus* R2-SPc | original | *psbAI* (1**efe*) | $P_{psbA1}$ | 7 | N.R. | N.R. | (Wang et al. 2000) |
| 10 | *Synechococcus* | original | *psbAI* (1**efe*) | $P_{psbA1}$ | 451 | - | yellow-green | (Takahama et al. 2003) |
| 11 | *Synechococcus* | original | *NSI* (1**efe*) | $P_{trc}$ | 140 | + | blue-green | This work |
| 12 | *Synechococcus* | optimized [(b)] with His-tag | *NSI* (1**efe*) | $P_{trc}$ | 140 | + | blue-green | This work |

| ID | Host and other genetic modifications | *efe* gene and modifications | Expression plasmid/ integration site | Promoter | Max. reported productivity ( $\mu L*L^{-1}*h^{-1}*OD^{-1}$ ) | Stability | Phenotype | Reference |
|---|---|---|---|---|---|---|---|---|
| 13 | *Synechocystis* | optimized [(a)] 3X-FLAG tag | *slr0168* (1**efe*) | $P_{psbA}$ | ≈370 | + | blue-green | (Ungerer et al. 2012) |
| 14 | *Synechocystis ΔpsbAII* | optimized [(a)] 3X-FLAG tag | *slr0427*, *slr0168* (2**efe*) | $P_{psbA}$ | ≈570 | + | blue-green | (Ungerer et al. 2012) |
| 15 | *Synechocystis* | optimized [(b)] His-tag | pDF-trc-EFEh | $P_{trc}$ | ≈170 | + | N.R | (Guerrero et al. 2012) |
| 16 | *Synechocystis* | optimized [(b)] His-tag | pDF-lac-EFEh | $P_{A1lacO-1}$ | ≈170 | N.R | N.R | (Guerrero et al. 2012) |
| 17 | *Synechocystis* | optimized [(b)] His-tag | pDF-pet-EFEh | $P_{petE}$ | ≈28 | N.R | N.R | (Guerrero et al. 2012) |
| 18 | *Synechocystis* | optimized [(b)] His-tag | pDF-coa-EFEh | $P_{coa}$ | ≈48 | N.R | N.R | (Guerrero et al. 2012) |
| 19 | *Synechocystis* | optimized [(b)] His-tag | pDF-smt-EFEh | $P_{smt}$ | ≈2 | N.R | N.R | (Guerrero et al. 2012) |
| 20 | *Synechocystis Δogdc/ Δssadh kgtP* (*ΔphaAB*) | optimized [(c)] His-tag | *slr0168*, *sll1981*, *slr0370* (3**efe*) | $P_{cpcB}$ | 858 | + | N.R | (Zhu et al. 2015) |
| 21 | *Synechocystis Δogdc/ Δssadh* | optimized [(c)] His-tag | *slr0168*, *sll1981*, *slr0370* (3**efe*) | $P_{cpcB}$ | ≈360 | N.R | N.R | (Zhu et al. 2015) |
| 22 | *Synechocystis Δogdc* | optimized [(c)] His-tag | *slr0168* , *sll1981* (2**efe*) | $P_{cpcB}$ | ≈220 | N.R | N.R | (Zhu et al. 2015) |
| 23 | *Synechocystis Δogdc* | optimized [(c)] His-tag | *sll1981* (1**efe*) | $P_{cpcB}$ | ≈130 | N.R | blue-green | (Zhu et al. 2015) |

| | | | | | | | | |
|---|---|---|---|---|---|---|---|---|
| 24 | *Synechocystis* Δ*ssadh* | optimized [c] His-tag | *slr0370* (1**efe*) | $P_{cpcB}$ | ≈90 | N.R | blue-green | (Zhu et al. 2015) |
| 25 | *Synechocystis* | optimized [c] His-tag | *slr0168* (1**efe*) | $P_{cpcB}$ | 774 | N.R | N.R | (Zhu et al. 2015) |
| 26 | *Synechocystis* | optimized [c] His-tag | *slr0168* (1**efe*) | $P_{psbA2(6803)}$ modified | 518 | + | N.R | (Zhu et al. 2015) |
| 27 | *Synechocystis* | optimized [c] His-tag | *slr0168* (1**efe*) | $P_{psbA2(6803)}$ | ≈520 | N.R | N.R | (Zhu et al. 2015) |
| 28 | *Synechocystis* | optimized [c] His-tag | *slr0168* (1**efe*) | $P_{psbA2(7942)}$ | ≈350 | N.R | N.R | (Zhu et al. 2015) |
| 29 | *Synechocystis* | optimized [c] His-tag | *slr0168* (1**efe*) | $P_{rbc}$ | ≈650 | N.R | N.R | (Zhu et al. 2015) |
| 30 | *Synechocystis* | optimized [c] His-tag | *slr0168* (1**efe*) | $P_{psbD}$ | ≈450 | N.R | N.R | (Zhu et al. 2015) |
| 31 | *Synechocystis* | optimized [c] His-tag | *slr0168* (1**efe*) | $P_{psbA1}$ | ≈420 | N.R | N.R | (Zhu et al. 2015) |
| 32 | *Synechocystis* | optimized [c] His-tag | *slr0168* (1**efe*) | $P_{groESL}$ | ≈300 | N.R | N.R | (Zhu et al. 2015) |
| 33 | *Synechocystis* | optimized [c] His-tag | *slr0168* (1**efe*) | $P_{glnA}$ | ≈340 | N.R | N.R | (Zhu et al. 2015) |
| 34 | *Synechocystis* | optimized [c] His-tag | *slr0168* (1**efe*) | $P_{cI}$ | ≈390 | N.R | N.R | (Zhu et al. 2015) |
| 35 | *Synechocystis* | optimized [a] 3X-FLAG tag T7 TT, rbsV4 | *slr0168* (1**efe*) | $P_{psbA}$ | 718 | N.R | N.R | (Xiong et al. 2015) |
| 36 | *Synechocystis* Δ*psbAII* | optimized [a] 3X-FLAG tag | *slr0427*, *slr0168* (3**efe*) | $P_{psbA}$ | ≈550 | N.R | N.R | (Xiong et al. 2015) |

| | | | | | | | | |
|---|---|---|---|---|---|---|---|---|
| 37 | *Synechocystis* Δ*psbAII xylAB* (Δ*slr0168*) | optimized [(a)] 3X-FLAG tag | *slr0427* (1**efe*) | $P_{psbA}$ | ≈177 | N.R | N.R | (Lee et al. 2015) |
| 38 | *Synechocystis* | original | pDF-trc-o-*efe* | $P_{trc}$ | ≈230 | + | N.R | (Carbonell et al. 2016) |
| 39 | *Synechocystis* | optimized [(b)] | pDF-trc-sy-*efe* | $P_{trc}$ | ≈170 | + | N.R | (Carbonell et al. 2016) |
| 40 | *Synechocystis* (motile) | optimized [(d)] lambda *oop* TT | pVZ325-$P_{tac}$-efe | $P_{tac}$ | 500 | N.R | blue-green | (Kuchmina et al. 2017) |
| 41 | *Synechocystis* Δ*ogdc*/Δ*ssadh* Δ*ntcA* (unsegregated) | optimized [(c)] His-tag | *slr0168*, *sll1981*, *slr0370*, *sll1423* (4**efe*) | $P_{cpcB}$ | 2463 | N.R | N.R | (Mo et al. 2017) |
| 42 | *Synechocystis* Δ*ogdc*/Δ*ssadh* Δ*ntcA* (unsegregated) | optimized [(c)] His-tag | *slr0168*, *sll1981*, *slr0370* (3**efe*) | $P_{cpcB}$ | ≈1750 | N.R | N.R | (Mo et al. 2017) |
| 43 | *Synechocystis* Δ*ntcA* (unsegregated) | optimized [(c)] His-tag | *slr0168* (1**efe*) | $P_{cpcB}$ | 1572 | N.R | N.R | (Mo et al. 2017) |
| 44 | *Synechocystis* NtcA OE (Δ*phaAB*) | optimized [(c)] His-tag | *slr0168* (1**efe*) | $P_{cpcB}$ | ≈800 | N.R | N.R | (Mo et al. 2017) |
| 45 | *Synechocystis* | optimized [(e)] RBS-0 (SD), TT BB0014 | *slr0168* (1**efe*) | $P_{trc}$ | 103 | N.R | N.R | (Veetil et al. 2017) |
| 46 | *Synechocystis* | optimized [(e)] RBS-34, TT BB0014 | *slr0168* (1**efe*) | $P_{trc}$ | ≈135 | N.R | N.R | (Veetil et al. 2017) |
| 47 | *Synechocystis* | optimized [(e)] RBS-30, TT BB0014 | *slr0168* (1**efe*) | $P_{trc}$ | 195 | N.R | N.R | (Veetil et al. 2017) |
| 48 | *Synechocystis* | optimized [(e)] RBS-H, TT BB0014 | *slr0168* (1**efe*) | $P_{trc}$ | ≈30 | N.R | N.R | (Veetil et al. 2017) |
| 49 | *Synechocystis* | optimized [(e)] RBS-C9, TT BB0014 | *slr0168* (1**efe*) | $P_{trc}$ | ≈10 | N.R | N.R | (Veetil et al. 2017) |

| | | | | | | | | |
|---|---|---|---|---|---|---|---|---|
| 50 | *Synechocystis* | optimized [e] RBS-C10, TT BB0014 | *slr0168* (1**efe*) | $P_{trc}$ | ≈10 | N.R | N.R | (Veetil et al. 2017) |
| 51 | *Synechocystis* | optimized [e] RBS-C11, TT BB0014 | *slr0168* (1**efe*) | $P_{trc}$ | ≈10 | N.R | N.R | (Veetil et al. 2017) |
| 52 | *Synechocystis* | optimized [e] RBS-30, TT BB0014 | *slr0168* (1**efe*) | 3*$P_{trc}$ | ≈175 | N.R | N.R | (Veetil et al. 2017) |
| 53 | *Synechocystis* | optimized [e] RBS-30, TT BB0014 | *slr0168* (1**efe*) | 5*$P_{trc}$ | ≈175 | N.R | N.R | (Veetil et al. 2017) |
| 54 | *Synechocystis* | optimized [e] RBS-30, TT BB0014 | pVZ321-$P_{trc}$-Rbs30-efe | $P_{trc}$ | ≈90 | N.R | N.R | (Veetil et al. 2017) |
| 55 | *Synechocystis* Δ*glgC* | optimized [e] RBS-30, TT BB0014 | *slr0168* (1**efe*) | $P_{trc}$ | ≈120 | N.R | N.R | (Veetil et al. 2017) |
| 56 | *Synechocystis* Δ*glgC* | optimized [e] RBS-30, TT BB0014 | pVZ321-$P_{trc}$-Rbs30-efe | $P_{trc}$ | ≈105 | N.R | N.R | (Veetil et al. 2017) |

efe: ethylene forming enzyme; +: Stable; -: Unstable; N.R: Not Reported; TT: Transcription terminator; OE: over-expression; SD: Shine Dalgarno; (a): Optimized sequence from *P. syringae* pv. *Phaseolicola* as done in (Ungerer et al. 2012); (b): Optimized sequence from *P. syringae* pv. *phaseolicola* as done in (Guerrero et al. 2012); (c): Optimized sequence from *P. syringae* pv. *sesami* as done in (Zhu et al. 2015); (d) Optimized sequence from *P. syringae* pv. *phaseolicola* PK2 as done in (Kuchmina et al. 2017) (e) Optimized sequence from *P. syringae* pv. *phaseolicola* as done in (Veetil et al. 2017).
